# Beyond individual barriers and facilitators: Digital interventions to address diabetes in urban Ghana

**DOI:** 10.1177/20552076251349705

**Published:** 2025-07-28

**Authors:** Ethan Gray, Ann Blandford, Samuel Amon, Publa Antwi, Vida Asah-Ayeh, Raphael Baffour Awuah, Leonard Baatiema, Sandra Boatemaa Kushitor, Hassan Haghparast-Bidgoli, Hannah Maria Jennings, Irene Akwo Kretchy, Daniel Strachan, Megan Vaughan, Edward Fottrell

**Affiliations:** 1 204274 Institute of Global Health, University College London, London, UK; 2 Department of Computer Science, 4919 University College London, UCLIC, London, UK; 3 Noguchi Memorial Institute for Medical Research, 58835 University of Ghana, Accra, Ghana; 4 66134 Department of Health Sciences, University of York, York, UK; 5 118922 Regional Institute for Population Studies, University of Ghana, Accra, Ghana; 6 525891 Vital Strategies, New York, NY, USA; 7 Department of Health Policy, Planning and Management, 260088 School of Public Health, 58835 University of Ghana, Accra, Ghana; 8 Hull York Medical School, 66134 University of York, York, UK; 9 Department of Pharmacy Practice and Clinical Pharmacy, School of Pharmacy, 58835 University of Ghana, Legon, Ghana; 10 The Nossal Institute for Global Health, University of Melbourne, Melbourne, Australia; 11 170688 University College London Faculty of Social & Historical Sciences, Institute of Advanced Studies, London, UK

**Keywords:** Digital health, mHealth, community health, type 2 diabetes, non-communicable diseases, task-shifting, empowerment

## Abstract

**Objective:**

The prevalence of type 2 diabetes (T2D) and other non-communicable diseases (NCDs) in Ghana and other countries in sub-Saharan Africa (SSA) is increasing at a rate notably higher than the rest of the world. Consequently, there is an urgent need to develop low-cost community interventions for diseases including T2D in Ghana, with digital tools potentially empowering community members in prevention and management. This research aimed to identify effective strategies for leveraging digital tools to address T2D in Ga Mashie, Ghana, through community-driven empowerment and action.

**Method:**

This was a mixed methods study involving focus groups (N = 13), qualitative interviews with community representatives (N = 69) and two community workshops (N = 35 participants in each).

**Results:**

The focus groups and interviews identified strong facilitators for an individual-level digital intervention focused on education; however, workshops highlighted that the community wants greater access to in-person education and healthcare services in Ga Mashie, limiting the likely impact of an individual digital intervention.

**Conclusion:**

Our findings challenge the widespread assumption that digital interventions should be targeted at the individual; rather, digital tools might be used to empower community leaders in Ga Mashie with training and clinical guidance to function as healthcare agents, scaling-up the delivery of education and screening services to their broader community. This suggests a novel system-level strategy for designing community-based, empowerment-focused digital health interventions that reflect the practices and values of community members, though further work is needed to validate this approach.

## Introduction

The prevalence of non-communicable diseases (NCDs), including type 2 diabetes (T2D), in sub-Saharan Africa (SSA) is increasing at a rate notably higher than the rest of the world^1^ with more than two-thirds of the global rise of T2D occurring in this context.^2^ This pattern is reflected within Ghana, with the estimated number of cases of T2D having nearly tripled over the past 20 years.^2^ While T2D has been estimated to affect between 2.8% and 4.0% of the Ghanaian population,^3^ barriers to diagnosis suggest the actual prevalence may be more than double this.^1^

This rapid increase across SSA has meant that already-overburdened health systems are unable to cope with the burden of T2D.^1^ This is largely a result of a historical prioritisation towards managing infectious diseases in these contexts, resulting in inadequate guidelines, healthcare personnel, formal training, diagnostic equipment and medications to effectively manage NCDs.^1,4^ As a result, NCDs such as T2D are emerging as the leading causes of death across SSA, overtaking infectious diseases.^1^ These diseases affect poor communities disproportionately, exacerbating poverty and creating long-term psychosocial challenges.^5^ Thus, intervention is urgently required to meet the United Nations Sustainable Development Goal of reducing premature mortality from NCDs by 2030.^6^

There is already extensive literature on digital interventions that empower individuals through increasing access to education and supportive guidance. Telemedicine interventions have been used to successfully support patients with T2D to control blood glucose levels and engage with their healthcare system.^7,8^ Significant evidence supports the value of Short Message Service (SMS)-based interventions to deliver supportive advice and reminders to individuals with T2D to improve the capacity for self-care and medication adherence.^9–11^ This evidence affirms the potential of digital resources to reduce reliance on existing healthcare services by empowering individuals to function as their own healthcare agents.^12^

This study aimed to investigate potential strategies for leveraging digital resources to support and empower people in Ga Mashie, an urban community in Accra, Ghana, in preventing and managing T2D. Aligned with prevailing literature, our initial assumption was that digital strategies would facilitate empowerment by targeting the individual, with our first research question exploring the feasibility of an individual-level digital intervention in this context. Further, grounded in principles of participatory learning and action (PLA),^13^ as described below, this study engaged the target community to co-identify intervention strategies that would address their needs through preferred approaches. Thus, this study aimed to define effective strategies for leveraging digital tools to empower Ga Mashie residents to address T2D in a way that fundamentally supports the preferences and values of the community.

## Contextual background

This study was conducted under the larger ‘CARE: Diabetes in Ghana’ project, which seeks to understand and address the burden of T2D in Ga Mashie.^14^ With a population of about 80,000,^15^ Ga Mashie (consisting of neighbouring suburbs James Town and Ussher Town) was once the economic centre of Ghana's capital city, but recent decades have seen the community suffering from a declining socioeconomic status, threatening the population's wellbeing. Despite this, Ga Mashie is defined by a strong collectivist sense of community with respected community leaders.^16^ Working with the community, the CARE project aimed to define the burden, context and community challenges around T2D in Ga Mashie and empower the Ga Mashie community towards designing and implementing a community health intervention to address its key challenges around T2D. Quantitative methods and findings related to the first aim are reported elsewhere.^14,17,18^ In brief: a cluster survey of adults was conducted in Ga Mashie, targeting 959 eligible households. The survey achieved a 67% response rate, providing details for 854 individuals representing 644 households. The survey unveiled a range of non-communicable disease risk factors: 47.2% for alcohol consumption (95% confidence interval (CI): 43.7–50.8), 73.3% for insufficient physical activity (95% CI: 69.1–77.1), 28.9% for unhealthy snack consumption (95% CI: 24.5–33.7), 35.1% for obesity (95% CI: 31.3–39.1) and 74.5% for central obesity (95% CI: 70.8–77.9). Diabetes affected 8.2% of the population aged ≥25 (95% CI: 6.4–10.5).

To keep the survey to a reasonable length, data on mobile phone ownership was not gathered. However, other sources indicate that ownership of mobile phones is high in Ghana, with over 99% of adults aged 16 to 64 possessing a smartphone in 2022, rising to nearly 100% by 2024.^19^

The study reported here focused on whether there was a role for digital tools in addressing the burden of T2D and, if so, what the potential role(s) for digital interventions were. We explored the digital context of Ga Mashie to ideate effective strategies for leveraging digital to empower the community. Specifically, we explored the digital context at the individual and health-system levels to evaluate the barriers and facilitators that determine the feasibility of a digital intervention that empowers the individual. We further engaged the target community to co-identify their needs in relation to a digital intervention, identifying requirements for designing digital interventions that are not only feasible but also aligned with the community's values.

This study was guided by CARE's tenets of facilitating empowerment through community participation in the problem- and solution-identification processes. Grounded in the principles of PLA,^13^ CARE aimed to facilitate the community in identifying their main challenges around T2D and co-designing strategies tailored to their specific context. By positioning community members as knowledge experts throughout the research process, CARE aimed to design interventions that would ensure acceptance, uptake and engagement by community members by aligning with their preferences and values.

Extending this philosophy, this study explored beyond the basic infrastructural constraints that determine the feasibility of digital interventions, aiming to understand the community's requirements around a T2D digital intervention. Hence, this research offers reflections on key assumptions about the design of digital interventions in the global south, recognising the importance of empowering and engaging users throughout the design process.

## Research background

**Figure 1.**
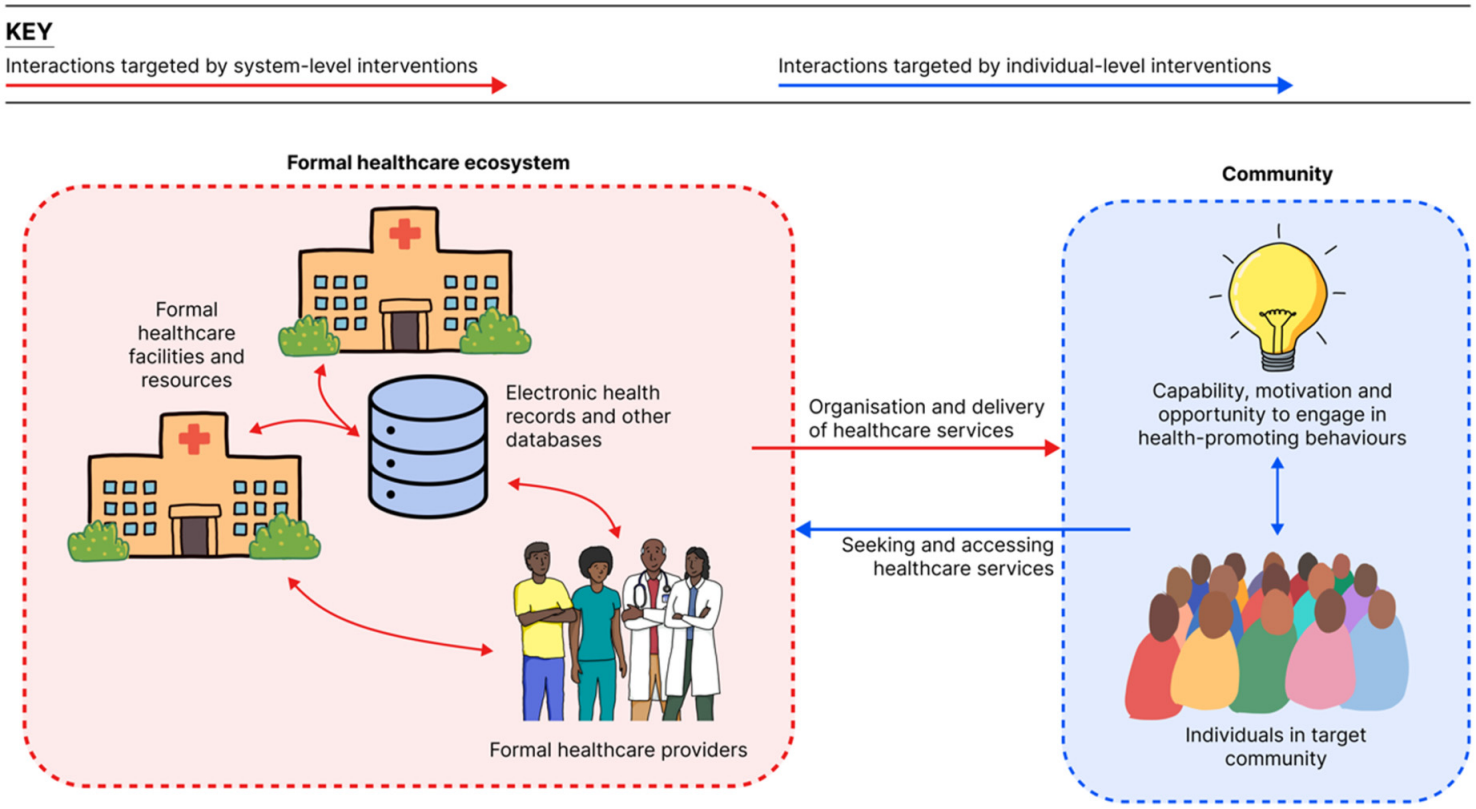
Diagram showing individual- and system-level digital innovations.

Digital resources have garnered significant global support as means to overcome challenges contributing to T2D. They potentially address individual and structural barriers perpetuating healthcare inequity in rural, isolated or poorer communities with limited access to formal health services.^20^ As noted above, the prevalence of mobile phones in Ghana is high and rapidly growing, suggesting that mobile health interventions offer a feasible route for improving healthcare and wellbeing.^21^ Recognising the access to these digital resources and their capacity to improve healthcare delivery, it is important to explore how digital resources may be leveraged to address T2D, with individual- or system-level strategies being adopted in prior studies (Figure 1).

### Individual-level strategies

Individual-level strategies improve the way that individuals engage with a healthcare system to maximise their benefit from that system, ensuring that the individual is more empowered in relation to their broader healthcare ecosystem^22^ (Figure 1). A common example is telehealth services, whereby existing health services, previously delivered in person, are delivered digitally through mediums such as mobile phones.^23^ Telehealth strategies enable individuals in remote or isolated communities, with limited access to in-person services, to access such services remotely, overcoming access barriers.^24^ Such strategies have been used to provide patients with T2D with remote healthcare consultations, supporting them in effectively controlling blood glucose levels and engaging with their healthcare system.^7,8^

Individual-level strategies may also mitigate reliance on existing healthcare services by empowering individuals to function as their own healthcare agents.^12^ These strategies seek to improve everyone's capacity, opportunity and motivation for self-care behaviours, empowering individuals to manage common complications.^24,25^ Consequently, engagement with less accessible formal healthcare services is streamlined to more severe and complicated cases.^26^ For T2D, such strategies have focused on the provision of individual-level health education, due to its capacity to improve knowledge and performance of healthy behaviours around T2D in contexts like Ghana.^27–31^

Aligned with this, there are many examples of digital tools, commonly available on mobile phones, successfully being used to deliver T2D education at an individual level.^32^ Such tools offer the capacity to deliver education at scale without heavily relying on human resources or direct access to healthcare facilities, potentially offering significant value in contexts like Ghana.^33^ A meta-analysis on using SMS to deliver supportive education to individuals with T2D observed that such interventions improve the capacity for self-care, reflected in lower blood glucose levels.^34^ Similar effectiveness has been observed in low–middle income contexts, such as the mDiabetes programme in Senegal, which provided SMS-based advice and reminders for patients with T2D, reducing reliance on T2D medication.^9^ Similarly, the StAR2D programme in Malawi and South Africa effectively employed text messaging to improve medication adherence and clinical outcomes, with participants stating that the text format addressed informational needs and increased perceptions of social support.^10,11^

### System-level strategies

System-level strategies focus on using technology to improve the capacity of a healthcare system to address the health needs of a population.^20^ These strategies target the organisation and delivery of healthcare services through more effective use of existing healthcare resources, indirectly improving the individual's access to healthcare and consequent wellbeing (Figure 1). These strategies use digital resources to complement and enhance existing modes of healthcare delivery, ensuring that digital does not function as a substitute for services that are familiar and acceptable to the individual.^20^ Thus, such strategies aim to collectively strengthen the existing healthcare ecosystem so that it may better reach individuals in a community through established and familiar avenues.^21^

Within the global south, effective system-level innovation may involve scaling up health resources to increase accessibility of healthcare services. Task-shifting approaches have emerged as effective and viable strategies to rapidly scale-up healthcare provision in resource-constrained contexts.^35^ Task-shifting involves the redistribution of health tasks amongst workforce teams to enable basic services, commonly performed by doctors or nurses at clinics or hospitals, to be performed by lower-skilled workers at the community level.^36^ Within T2D, task-shifting approaches emphasise upskilling community volunteers as health workers to provide health-promotion services, such as education, screening, and measurement of blood glucose levels, in their community, making these services more accessible.^35,37^ These workers are also trained to refer patients to appropriate services for more specialised intervention when required. In this sense, they function as gateways to formal healthcare, supporting patients in accessing appropriate care for their condition while simultaneously minimising the burden on specialised services.^38^ Such approaches have been shown to improve the utilisation of healthcare services and patient outcomes and collectively empower communities to adopt active roles in their wellbeing.^35^

Digital tools present significant value in supporting task-shifting approaches. Firstly, digital technologies can assist in training community health workers, making ongoing learning more accessible through remote formats such as video lectures or mobile applications.^39–41^ During COVID-19, Ghanaian formal healthcare providers were trained using desktop computers on the delivery of a T2D self-management education programme, with providers describing the medium as effective for knowledge acquisition and positive to use.^42^ Technologies can also increase access to supportive clinical guidance for community workers through digital decision-support agents or remote consultations with specialised workers,^39,40,43,44^ enabling community workers to make informed care decisions in cases beyond the scope of initial training and perform clinical roles effectively within their community.^45^

### Networks of practice and other digitally enabled interventions in Ghana

In 2011, the Novartis Foundation piloted a telemedicine intervention connecting community health workers to doctors and nurses via 24-hour call centres to offer advice on the management of patients at the community level.^46^ As the intervention successfully empowered community health workers to resolve cases in their communities, it was scaled up nationally. A similar intervention called the Community-based Hypertension Improvement Project implemented by FHI 360 with the Ghana Health Service and funded by Novartis was subsequently developed for hypertension.^42^ Conducted in Lower Manya Krobo municipality, the intervention used mobile devices to connect community nurses and other multi-sector partners (such as local shop owners or pastors) with physicians to receive ongoing remote training and supervision on delivering screening and referral services in their community.^47^ The intervention effectively enabled the scale-up of screening services without significant resource investment and improving hypertension control rates both immediately and at the 12-month follow up.^47^

NGOs PharmAccess and Luscii, in collaboration with the University of Ghana, subsequently adapted this approach to the management of NCDs in Ghana with the development of NCDCare, a mobile app that aims to assist in the management and treatment of T2D and hypertension through symptom tracking and information provision.^48^

Building on these projects, and to optimise access to quality and equitable healthcare, Ghana adopted the ‘Network of Practice’ (NOP) approach from January 2023, as the new policy direction and framework for primary healthcare in Ghana,^49^ with implementation being piloted within two urban districts outside Accra. The policy recognises the role of digital health, through telemedicine, to address access-based barriers to accessing quality care through collaboration across networks of providers at various tiers of care in each district, enabling effective coordination of care between community-based facilities, primary-level clinics, and more specialised district hospitals.^49^ NOP emphasises the remote provision of information and clinical decision support to lower tiers of care through digital mediums to improve the services that can be offered at the primary and community levels.^49^ Simultaneously, NOP encourages effective referrals across levels of care so that patients are directed to the most appropriate facility for their needs. Through this, community services can function as accessible gateways into the care system, improving access to and utilisation of appropriate healthcare services.

In parallel, many non-governmental health organisations have incorporated mobile phones as mediums to increase access to trusted advice from healthcare providers. Such initiatives emerged around 2013, with the launch of Vodafone's ‘Healthline 255’ initiative.^50^ This service allows users to dial a toll-free number where they can receive health promotion and healthcare advice from clinicians. Healthline 255 claimed to have played a crucial role in managing the Ebola and COVID-19 outbreaks in Ghana.^50,51^ In addition, phone-based consultations have been integrated into the service delivery model offered by a private health insurance provider, BIMA, since 2018.^52^ Through such services, subscribers can access information on the prevention and management of common NCDs or specific to conditions they have been diagnosed with.

### Favouring individual approaches

While individual- and system-level digital strategies function complementarily to improve a community's wellbeing, individual-level approaches are increasingly favoured for empowering communities over their health.^12^ There are many reports of digital behavioural change interventions (DBCIs), whereby digital tools aim to improve an individual's wellbeing by increasing their engagement in healthy behaviours, empowering individuals as agents over their health.^53^ Such strategies position individuals as target users, aiming to address individual-level challenges that limit their motivation, capability and opportunity to engage in target behaviours.^53^ Individual-level strategies such as these have attracted widespread interest in the global South for their potential to improve patient outcomes, create a greater sense of autonomy and empowerment, and mitigate the challenge of solving complex entrenched systemic challenges.^23,39^ This has resulted in such strategies being perceived as simpler and more efficient routes to uplifting the wellbeing of communities in contexts such as Ghana than system-level approaches.

Recognising the value of individual-level digital interventions, it is important to explore the factors that determine their feasibility. On an individual level, digital innovations are primarily limited by individuals’ access to digital resources, such as mobile phones and internet connectivity, and their digital literacy in the context of health.^54,55^ The penetration of digital infrastructure in Ghana suggests that the target context should be exempt from such barriers, resulting in individual-level interventions being feasible.^19^ On a broader level, the presence of regulatory, financial, and leadership support is a vital determinant of the success of digital interventions.^55^ Research highlighting these factors^54,55^ suggests that in contexts where people have sufficient technology access and literacy, and where institutional frameworks support implementation, individual-level digital interventions can be a promising way to address healthcare challenges.

### Designing for unique user needs: A focus on PLA

Even if the above feasibility constraints are satisfied, effective individual-level digital interventions must be designed for the needs of the target user. Digital interventions need to deliver solutions that will improve clinical outcomes without placing additional strain on the individual or their healthcare system.^12^ Digital interventions must also be acceptable to the user to ensure effective uptake and continued engagement.^56^ This can be achieved by ensuring interventions fit the user's value system, are not burdensome to use, and are perceived by the user as likely to achieve their desired purpose.^56^ This requires actively engaging target users along all stages of the design process through human-centred strategies, such as co-design and ethnography.^57^ Through these processes, one can identify and design for the user's needs and preferences to ensure that the intervention is helpful, effective, learnable and likeable.^58^ Many digital interventions experience low utilisation despite their promising value from a research perspective because of individual preferences being overlooked in the design process.^54^

By applying the principles of PLA to intervention research and development, one can ensure that the user requirements are prioritised. PLA is a dynamic and inclusive philosophy that may be applied to design community-based interventions.^59,60^ It requires the active involvement of the target community throughout the research and development processes to aid empowerment and ownership among participants and ensure proposed solutions align with the community's values and preferences.^13^ PLA has been applied to intervention strategy development through a four-stage framework: first, conducting participatory assessments to understand the community's needs, resources and challenges; second, engaging stakeholders through workshops, discussions and feedback to co-create intervention strategies that align with the community's needs; third, community-driven implementation of the target intervention strategies; fourth, informally evaluating the progress and impact of the intervention strategies and adjusting guidelines accordingly.^60^ Through applying this to the design of digital interventions, one can ensure that interventions are not only feasible, but also likely to be accepted and adopted by the target users. In this article, the outcomes of the first two stages of PLA, focusing on the digital, are reported.

In the first stage, interviews and focus group discussions explored the digital context of Ga Mashie to identify key barriers and facilitators that would determine whether an individual-level intervention would be feasible. In the second stage, community engagement workshops were conducted by the CARE team to co-design solution strategies with community representatives to address their key challenges. These workshops included evaluating the community's preferences and values relating to the design of a digital intervention.

## Research stage 1: Exploring feasibility within the digital context

### Aim

This stage of research aimed to identify the key barriers and facilitators that would determine the feasibility of an individual-level digital intervention in Ga Mashie.

### Methods

The CARE project conducted three qualitative data-gathering activities in 2022 and 2023 aiming to understand the ecosystem of Ga Mashie in relation to T2D (Table 1). Data were gathered by trained research assistants who were fluent in the local languages (Ga and Twi) as described by Baatiema et al.^61^

**Table 1.** Overview of the three qualitative activities that comprised this research stage.

	Activity 1	Activity 2	Activity 3
**Qualitative method**	13 Focus group discussions	39 Semi-structured interviews	30 Semi-structured interviews
**Participants**	Community members divided by T2D diagnosis, sex and age group	Healthcare providers working in the community, including formal doctors, nurses, and pharmacists; faith and herbal healers; and community health workers	Policymakers and NGOs operating in Ga Mashie and broader Accra
**Problem context aims**	Understand the attitudes, experiences and behaviours of the community in relation to T2D	Understand key systemic barriers around T2D management by healthcare services	Understand the institutional and regulatory ecosystem around T2D
**Digital-specific aims**	Understand community members’ access and behaviour around digital technologies, both generally and in relation to T2D	Explore the uptake and role of digital technologies within a healthcare context	Explore the systemic factors driving or hindering digital use in relation to T2D

T2D: type 2 diabetes.

Data were gathered in November and December 2022. These data were analysed thematically in May–July 2023, as described in more detail below.

The first data gathering activity involved 13 focus group discussions (FGDs) with community members. As described by Baatiema et al.,^61^ participants were recruited purposefully to represent different sexes, age groups and experiences of T2D. Focus groups were convened by sex, age and diagnoses of T2D (see Table 2), to explore the attitudes, experiences and behaviours of participants in relation to T2D. ‘Youth’ were defined as individuals aged 18 to 24 while those aged 25+ were grouped for the FGDs. Focus groups were convened in convenient local meeting spaces.

**Table 2.** Focus group dIscussion participants – overview.

Category	Number of participants	Location
Female youth	9	Ussher Town
Female youth	8	James Town
Females with diabetes	5	James Town & Ussher Town
Females with diabetes	7	Ussher Town
Females with diabetes	5	James Town
Females without diabetes	8	James Town
Females without diabetes	9	Ussher Town
Male youth	8	Ussher Town
Male youth	8	James Town
Males with diabetes	8	Ussher Town
Males with diabetes	6	James Town
Males without diabetes	9	Ussher Town
Males without diabetes	8	James Town
**Total:**	**98**	

The second data gathering activity involved 39 semi-structured interviews with healthcare providers in the community, including doctors, nurses and pharmacists; herbal and faith healers; and community health workers, to identify key systemic barriers around the management of T2D by healthcare services. Again, participants were recruited purposively to ensure broad representation of perspectives and experiences across the different kinds of healthcare providers. Interviews were conducted in private spaces convenient to participants.

The third data gathering activity involved 30 semi-structured interviews with policymakers and NGOs operating in Ga Mashie and broader Accra, offering insights about T2D from institutional and regulatory perspectives. Again, recruitment was purposive, aiming to recruit participants with varying roles and levels of expertise.

Each of these data-gathering activities included digital-specific explorations. The first explored individuals’ access and behaviour around digital technologies, both generally and in relation to healthcare. These elements explored individual-level feasibility factors around digital access, literacy and acceptability within a healthcare context. The second and third probed the role of digital technologies within the healthcare system context, exploring how digital technologies are used within Ga Mashie's health system to support T2D management. These health-system-level explorations evaluated the capacity and direction for digital technologies to be feasibly integrated into the existing healthcare environment, based on systemic support and existing successful integrations.

First, EG analysed qualitative data from activity 1 to explore the digital context of Ga Mashie at the individual level. The analysis was regularly discussed in detail with AB as it progressed. To isolate relevant sections from the large datasets, transcripts were initially searched using digital terms (‘phone’, ‘computer’, ‘digital’, ‘techn’ and ‘data’). Each returned section was then explored to extract relevant information, noting observations under bottom-up emerging themes. Insights were separated into those relating to the digital overview (digital access and digital behaviours) and specific elements of the digital health context (information-seeking, education, self-management and telemedicine), with associated subthemes. Transcripts were then searched for keywords related to health-specific themes (‘info’, ‘learn’, ‘educat’, ‘message’, ‘manage’), gathering insights from each instance to provide contextual depth to the initial observations. Insights were organised using an affinity-mapping approach, iteratively grouping insights under the established themes. Insights were also colour-coded in relation to the sphere to which the insight pertained (i.e. individuals, the community, or interactions between individuals and healthcare services). This visual encoding was used to highlight digital behaviours that emerged from the community versus those at a healthcare-system level.

This analysis process was repeated with data from activities 2 and 3 to explore the digital context of Ga Mashie at a healthcare-system level. As above, transcripts were searched using broad digital terms (‘phone’, ‘computer’, ‘digital’, ‘tech’ and ‘data’), extracting observations and organising them using an affinity map under identified themes. Themes included the general digital context, patient-focused innovation (patient–provider interactions, telecounselling and community-based care), and healthcare-organisation innovation (management of patient data and provider support). Following this, transcripts were searched for keywords relating to these themes (‘educat’, ‘track’, ‘share’, ‘refer’, ‘network’, ‘manage’ and ‘tele’) to provide further depth to each theme. As above, the initial analysis was conducted by EG in close collaboration with AB, and insights were colour-coded in relation to the stakeholders driving and benefitting from digital integration to understand how digital intersects at the individual and health-system levels.

The main insights from these investigations were collated to define the barriers and facilitators of an individual-level digital intervention for T2D in Ga Mashie.

### Results

The results highlight several facilitators within Ga Mashie's digital ecosystem about a digital intervention for T2D. Given the high penetration of mobile phones as information mediums in the community, there is potential to harness these technologies to support information-seeking. Furthermore, existing efforts to leverage mobile phones for information delivery in Ghana highlight this as an acceptable and familiar approach, facilitating adoption. From a health-system perspective, while digital tools are relatively unintegrated in the healthcare system, there is strong support for harnessing digital tools to scale up and improve community health services.

One barrier to uptake was identified, namely the cost of smartphones and potentially blood glucose testing equipment.

#### There is widespread adoption of mobile phones as information mediums at the community level

Research revealed broad access to mobile phones in Ga Mashie, with existing roles revolving around information dissemination in the community. Most participants reported ownership and regular use of mobile phones for text-based communication between community members. This communication is largely goal-directed, revolving around sharing local news or organising in-person community gatherings. A community member without T2D noted: ‘*It helps in information dissemination. For instance, during organization of events, messages are sent through phone*.’ (female youth FGD), while another participant without T2D stated that ‘*there's no way you can effectively work without a phone. It serves as a critical means of communication between workers through WhatsApp so you need to have a smart phone. All information passes through phone. Another advantage is that it helps me in social interactions like communicating with friends and managing social groups*.’ (female youth FGD). Mobile phones also offer community members access to news and information from outside the community, which can then be distributed through this medium, as highlighted by a community member with T2D: ‘*The phone is very helpful to us. […] I check the news too and I get information that I share with people*.’ (males with T2D FGD). This external information had previously been accessed primarily through television and radio, but the specificity and autonomy offered by mobile phones for information-seeking has resulted in a shift towards their use.

In line with the dominant use of mobile phones as informational mediums, the community use mobile phones to seek and access specific trusted information in relation to health conditions such as T2D. A community member without T2D noted: ‘*Through my phone, I get some information on how to exercise when I wake up in the morning, what to eat, and how to live healthy*.’ (Jamestown youth FGD). Participants search the internet, citing sources such as Wikipedia and Google, to clarify specific informational gaps about conditions, with such behaviour most prevalent amongst youth and people diagnosed with T2D. A young community member without T2D noted that ‘*mobile phones help because you can search for knowledge about sicknesses you do not understand to get meaning*’ (female youth FGD). Similarly, an elderly community member with T2D stated that ‘*if something happens to me, my children usually Google it and tell me what to do and what not to do*’ (females with T2D FGD). Mobile phones enable people to seek trusted and problem-specific information in a way that is accessible and immediate, allowing people to resolve confusion around information from other accessible sources without needing to engage with formal healthcare professionals. This was highlighted by three young community members without T2D noting that ‘*you will search [the internet] and it will give you the facts*’ (male youth FGD), ‘*I trust phones because any time you want to search, you can get information that you can trust about that particular illness right in that moment*’ (female youth FGD), and ‘*I trust two avenues: phones and doctors*’ (female youth FGD). However, as online information-seeking is more common amongst the youth and those with T2D, there is less uptake of these resources as informational tools amongst the older population undiagnosed with T2D. Considering that this population has a greater risk of T2D and more limited engagement with healthcare providers, there is an opportunity to leverage the educational potential of mobile phones to address the informational needs of this group.

#### Private organisations and individual providers are using mobile phones to empower patients through information

Some study participants reported receiving health information through subscribing to private providers. For example, a community member without T2D noted that ‘*Vodafone sends a short code for you to dial if you want to get health information*’ (females without T2D FGD). Users reported accessing information on the prevention and management of common NCDs (‘*We receive some text messages on our phones informing us of healthy daily diets and fruits to eat*’ (Jamestown youth FGD)) or specific to conditions that the user has been diagnosed with (‘*I receive a message on my cell phone from BIMA Health Care about diabetes, educating me about what to do because I am a diabetic patient*.’ (females with T2D FGD)).

Within the public healthcare system, some providers reported using mobile phones to improve patient–provider interactions. For example, they use SMS to offer supportive real-time advice or offer details about referrals to other providers or test results. As a pharmacist remarked, ‘*sometimes, some [patients] come, do their test and then when the results are ready, we may send it to them with information. But this is only for those who have asked for that service to be rendered for them. We just transmit the information to them*’*.* Providers noted value in this medium from the perspectives of both service provision and patient outcomes, highlighting that making direct interactions more accessible ensured greater adherence to treatment and more effective care. Notably, however, as remote care has not been incorporated into the public healthcare model, such services have limited support. A medical doctor noted that ‘*there is no formal system. If [patients] don’t understand something, we can send this information to you. [Using SMS and WhatsApp] is an innovation that we use because it is a way of helping clients*’. However, their organic emergence attests to their perceived value by agents within the healthcare system, suggesting such strategies may be feasible if effective financial and management support are secured through policy.

#### Emerging mobile apps aim to tailor care to the individual

In line with empowering individuals through access to reliable health advice, there has been a growth of mobile apps that aim to assist individuals with self-managing health conditions through symptom tracking and facilitating healthcare seeking. Policymakers noted that such technologies gained momentum in Ghana in response to the COVID-19 pandemic, aiming to increase the competency of individuals in managing their own wellbeing while simultaneously streamlining patient engagement with formal healthcare services. As a policymaker noted in activity 3, ‘*some people can download health, or dietician, or training apps on their phones … which helps you with controlling diabetes*’.

While such interventions were perceived to offer significant benefit to both patients and healthcare systems, participants noted that socioeconomic barriers limit their uptake across Ghana. Users are still responsible for measuring their blood glucose levels, which requires either upfront investment in these instruments for home use or costly regular visits to healthcare facilities. Furthermore, they need access to smartphones which limits value to the more affluent members of the population (‘*[These apps] come with technological challenges, because they work on smart phones and not all patients are privilege or have access to smart phones*’ (Policymaker)). As described by a policy maker, ‘*if I go to the community and [a community health worker] checks my sugar levels, my doctor gets access to that information, so if you are my doctor you can check these things at the community level, without coming to the facility. [My doctor] can call me and advise me and say that maybe my sugar is high today or it is low so this is what I must do.*’ Through this, the app may benefit individuals and healthcare providers without placing additional burdens on individuals with T2D.

#### Concluding feasibility evaluations of an individual-level digital intervention

Data on the digital ecosystem of Ga Mashie suggests that digital tools may be feasibly employed to upscale the community's access to education around T2D. Mobile phones were identified as widely accessible resources for active information-seeking to address gaps in understanding, with high trust and acceptance of information resources. Access to these resources is high and the community are also highly literate and disposed towards using these mediums for accessing health information. These represent strong individual-level facilitators for a digital T2D education intervention to be used by individuals in Ga Mashie.

The feasibility of education interventions is also reflected in the prior implementation of such strategies at a health-system level. There have been many attempts by health organisations to leverage the community's digital health-seeking behaviour by directly delivering trusted education and clinical guidance to individuals through mobile phones. However, the study highlighted socioeconomic barriers around population-wide smartphone access and paid healthcare services, which has limited the uptake of such innovations. This suggests a need for digital educational interventions that utilise more accessible mobile formats and better integrate with the existing health-seeking behaviours of the community. This may be achieved through human-centred design that engages the target users. Additionally, by securing greater financial support for such interventions through prioritisation in policy, such interventions may be made more affordable to less affluent populations. Thus, while barriers exist to such interventions, the dominance of individual-level facilitators suggests that such strategies may be feasible for addressing T2D in Ga Mashie. This was further investigated in the next stage of the research.

## Research stage 2: Inferring acceptability through community engagement

### Aim

The prior research stage indicated that an individual-level digital intervention, particularly one aimed at scaling up access to T2D education, would be feasible in Ga Mashie. This research stage aimed to identify the key requirements for such an intervention from a community perspective. Aligned with the principles of PLA, this stage engaged the community to define strategies for a T2D intervention that would address their needs, aiming to identify the preferences and values of the community in relation to a digital intervention.

### Methods

Conducted under the broader CARE intervention development research,^61^ this study drew on key aspects of PLA to coordinate community meetings, whereby the target community could be engaged in ideating community-led strategies to address their needs.

#### Community engagement meetings

Two community meetings, on 27 June and 4 July 2023, were organised with opinion leaders and community members of Ga Mashie to enable community participation in the identification of key challenges and solution strategies relating to T2D. These meetings were organised by, and involved, SA, VAA, HJ, PA, EG, SBK and RBA. The first meeting concluded the first stage of the intervention development cycle based in PLA principles. This meeting involved facilitating the community in collectively defining and prioritising their challenges and associated needs responding to the findings from the first stage.

The second meeting marked the beginning of the second stage of the intervention development cycle: supporting the community in collectively ideating feasible and acceptable solution strategies that could address the identified challenges.

#### Participants

Key residents of Ga Mashie were invited to attend the two meetings. Community members who had participated in the qualitative research phases were recruited, including lay community members with and without T2D as well as opinion leaders, such as market queens (women in the community who manage market-based trading of goods), faith healers, chief leaders, healthcare workers, and local policy makers. Approximately 60 community members were invited to attend the two meetings to ensure strong and representative attendance. As concern was raised over facilitating in-depth discussions with such a large group, attendees were divided into three smaller groups for in-depth discussion of the problems and solutions. In total, approximately 35 community members representing varied community roles participated in each meeting, with largely consistent attendance across both meetings.

Each workshop lasted a working day (9am to 4pm) including breaks and food. Each comprised short presentations from the research team interleaved with facilitated discussions (conducted in Ga). The discussions were recorded, with informed consent, and subsequently transcribed and translated for analysis. Both workshops took place at a respected community-based organisation's building at a location that was convenient and familiar to community members.

#### Extracting insights from the meetings

The CARE team collectively analysed the transcripts from the meetings, distilling key insights about the challenges, solution directions, and PLA process in general. Findings were used to infer the community's preferences around an intervention, including opportunities involving digital (the focus of this article). Through the co-design process and the solution strategies identified, this research explored how digital could effectively support these strategies in a way that is not only feasible but aligned with the preferences and values of the community.

### Results

**Figure 2.**
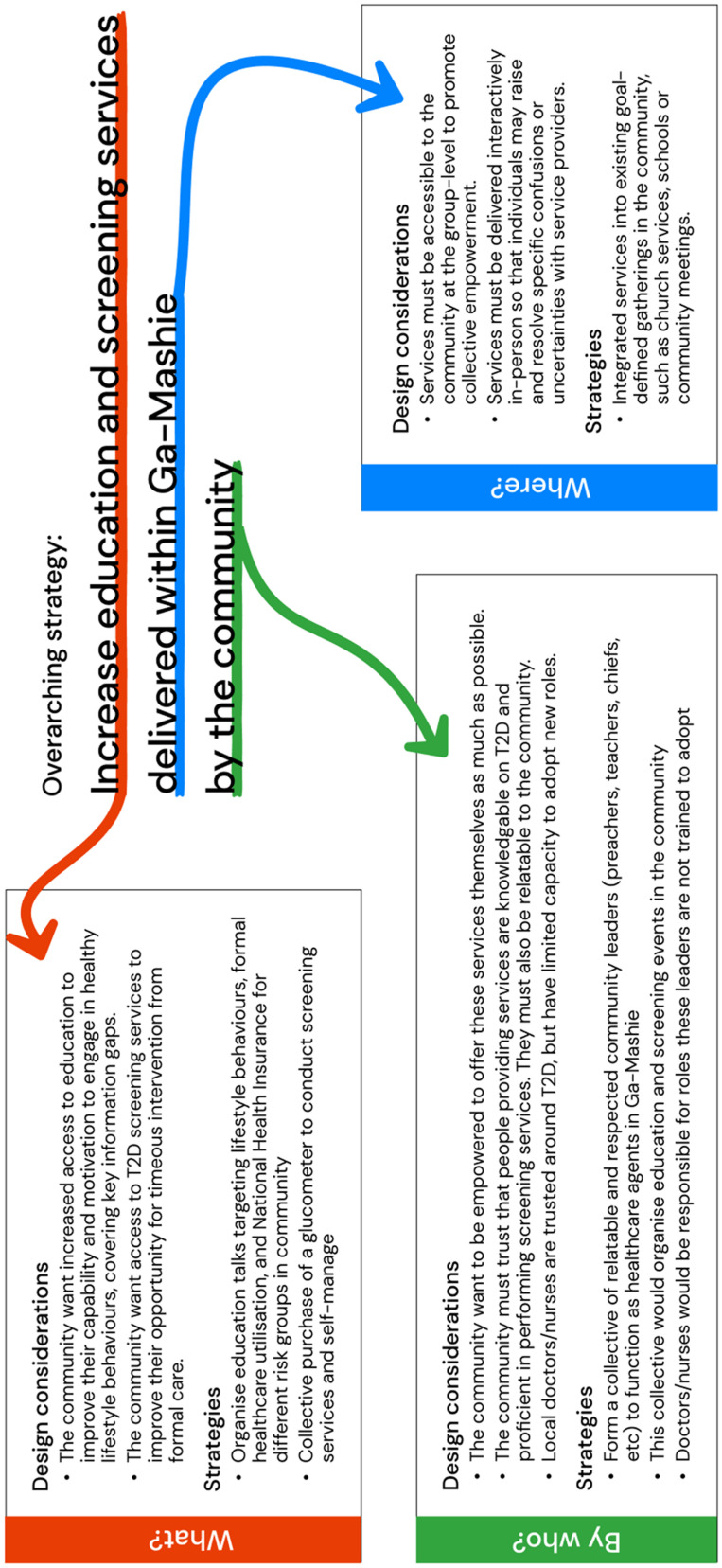
Primary solution strategies proposed by the community.

The community prioritised increasing access to education and T2D screening services within Ga Mashie to support positive lifestyle behaviours and effective engagement with formal healthcare services (Figure 2). Within this, education strategies should increase access to reliable information within the community so that community members can resolve information gaps. Strategies should also aim to increase the delivery of basic health-promotion services like testing blood sugar levels to screen for T2D within the community. To support these goals, the community proposed integrating education and screening services into existing social spaces and events in Ga Mashie. The final set of strategies aimed to empower the community to become self-reliant in the delivery of these services through the formation of a community health group that can adopt roles in organising and delivering these services.

#### Community education as the dominant intervention strategy

There was broad consensus that addressing key challenges requires access to reliable challenge-focused education. The community proposed that information that targets specific information gaps, is collectively accessed across the community and is provided in-person could help minimise the burden of T2D. The primary targets of such interventions would be lay community members, including those with T2D and those at risk of T2D.

The community highlighted the importance of tailoring education content to specific target groups and their associated information challenges. Firstly, emphasis was placed on providing early preventative education about T2D to children, focused on preventing rather than changing existing unhealthy behaviours. The second target group included older community members at greater immediate risk of T2D or with T2D, with education aiming to both motivate and increase the capability of this group to engage in behaviours to manage T2D.

The community emphasised that education strategies must utilise interactive, in-person approaches. As most community members possessed a basic understanding of T2D with varied information gaps, the need was for community members to be able to actively clarify information and query specific points about T2D. This would require access to a conversational information source, where individuals can raise specific confusions and receive tailored responses. To achieve this, the community proposed integrating education into existing social spaces that allow face-to-face interaction between education providers and community members. For example, emphasis was placed on integrating T2D education into schools for children or into churches and mosques for older adults. These mediums align with the community's preference for sharing, accessing and querying health information through human–human interactions with others in their community. Thus, to ensure trust, acceptance and engagement with an educational intervention, information must utilise these social pathways and settings to support active querying and engagement.

#### Upscaling community-based resources for T2D

Aligned with interventions aimed at upscaling interpersonal education delivery, other proposed strategies aimed to empower the community to adopt active roles in the provision of basic healthcare services for T2D. Such interventions would aim to integrate community members as community healthcare agents who can scale up access to services at the community level, extending the capacity of other community volunteers operating under the Ghanaian healthcare system. Through this, such interventions may address the broader community's opportunity and motivation barriers in regularly engaging with health services by increasing access to care. Additionally, such approaches would enable earlier detection and management for patients with T2D, reducing the burden of T2D on public health facilities.

In particular, the community proposed the collective purchase of T2D screening equipment (citing a glucometer specifically) to be used at community gatherings to screen individuals and evaluate T2D management, as observed in other research on T2D community interventions in the global South.^60^ As these services were cited as expensive and unpleasant to use regularly, empowering the community to provide these services themselves for free could address these challenges. Furthermore, such services equip the community with greater awareness about whether they have T2D or the effectiveness with which they are managing it, enabling more motivation and the capability to make informed decisions about their health behaviours.

#### Formation of a community health advocacy group to adopt healthcare roles

A crucial requirement of the above strategies is accessible and trusted human agents who can effectively deliver education and screening services within the community. Community members emphasised the importance of these in-person services being delivered by people the community trusted were well educated on T2D and performing and interpreting screening services, noting formal healthcare providers as ideal. However, the limited availability of these providers suggests that alternative more accessible community personnel need to be involved. This may include existing community health workers or health researchers who can adopt education roles due to their availability and trusted strong knowledge on T2D. Notably, however, attendees emphasised their responsibility in sharing the knowledge they had learnt with the broader community and adopting roles in managing T2D in Ga Mashie. The community proposed the formation of a community health advocacy group consisting of trusted and accessible community leaders such as teachers, pastors or community chiefs. This group would adopt healthcare roles in the community, such as organising and leading education and screening events alongside existing community healthcare workers. These leaders would also ideally be upskilled to provide education and screening themselves, overcoming the reliance on formal providers to perform these roles. To achieve this, leaders would need to be effectively educated through initial training and receive ongoing support from health experts in the form of supervision and advice. Thus, if these resources could be provided and reimbursement pathways defined, community leaders could be empowered to adopt roles as healthcare agents to provide education and screening around T2D.

#### Community preferences and values for a digital intervention

While community members did not directly reject the notion of delivering education and screening at an individual level through digital mediums, their core requirements indicated that this strategy, while feasible, was unlikely to satisfy their preferences and ensure effective uptake and sustained engagement. The community highlighted a preference towards services delivered through an in-person social medium, where their healthcare needs could be met through familiar human–human interactions. The emphasis on scaling-up community resources further suggests that the community value these existing and familiar modes of healthcare and desire an intervention that can effectively support, rather than supplement, them. Additionally, reflecting social values of Ga Mashie, the community highlighted a need for education and screening to be accessed collectively. This preference was underlined by a perception that community empowerment cannot occur at an individual level but must be achieved through joint participation and action. Thus, to address T2D in a way that supports the community's preferences, the role of digital cannot be to deliver the proposed intervention strategies at an individual level. Rather, digital should adopt a systemic supportive role, aiming to improve the organisation and delivery of existing in-person healthcare services at the community level.

In particular, the community proposed incorporating community leaders as agents who could adopt healthcare roles within their community. Thus, the digital may be more effective in supporting the upskilling and integration of these leaders into the healthcare ecosystem, rendering in-person healthcare services, such as screening and education, more accessible to the community in trusted face-to-face formats. This would reflect a more systemic digital innovation strategy, where the digital enacts change within the healthcare system rather than directly targeting the individual and how they intersect with their healthcare system. This strategy would seek to indirectly promote healthy behaviours amongst individuals in Ga Mashie, with this impact being mediated through empowered community leaders.

## Discussion

The second research stage revealed that an individual-level digital intervention for T2D would be unlikely to align with the community's preferences. This suggests that, despite technical feasibility, such an approach would have limited uptake, engagement and effectiveness. Conversely, an intervention focused on supporting in-person delivery of education and other basic health services would satisfy the community's needs, suggesting that a digital intervention at the health system level, empowering community leaders to deliver education and screening services for T2D, may be more appropriate.

There are many questions about the appropriateness of empowering community leaders as facilitators and educators for community healthcare that are beyond the scope of the research reported here. These include ethical considerations related to empowering established leaders with additional healthcare roles, questions of how to sustain and support such engagement over time, adapting existing approaches to data privacy and security and conducting economic and effectiveness evaluations of any implementation. These are discussed in the Future research section. As a starting point, however, we draw on our data to sketch a design possibility based on our findings, existing policy and priorities in Ghana, and prior research.

### A design sketch

Our findings suggest that digital tools, specifically mobile phones, may be used to upskill and integrate community leaders as health agents. This entails a novel strategy for designing digital health interventions focused on empowerment. Specifically, it aims to facilitate positive T2D behaviours in individuals indirectly by positioning leaders as intermediary target users, through which the digital intervention would impact the broader population. As noted, digital health interventions in low–middle income contexts have largely focused on addressing challenges on an individual level or through improving health infrastructure of the target community.^62^

**Figure 3.**
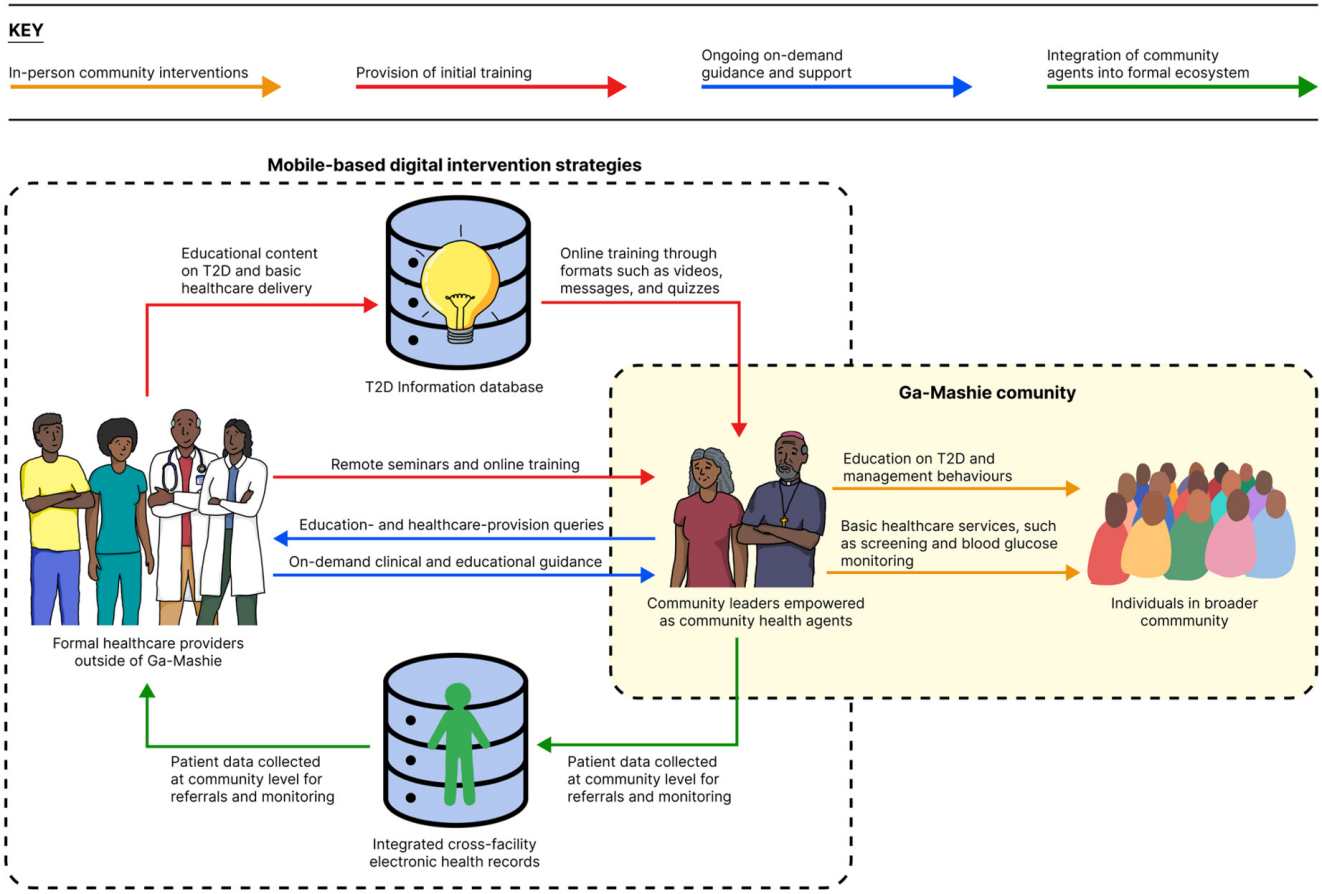
Proposed strategies for leveraging digital tools to empower community agents to address T2D in Ga Mashie.

The community's preferences and values emphasise the need for digital interventions at a broader healthcare-system level, with a specific focus on empowering community leaders to increase the delivery of community-based services, as illustrated in Figure 3. This reflects a task-shifting approach as outlined above. Implicit within this strategy is the value that Ga Mashie assigns to human–human interactions within healthcare, suggesting greater effectiveness, through greater acceptability and trust, of an intervention aimed at supporting in-person community health services.

Digital tools might be used to provide community leaders with initial training and ongoing clinical support to deliver education and basic screening services to function as community health agents. Additionally, in line with the NOP,^49^ digital tools could be leveraged to integrate these new community health agents into the broader healthcare ecosystem, ensuring that there is an effective flow of clinical information between the community and higher tiers of care.

#### Digital provision of initial training

The increasing digitisation of health training in Ghana indicates strong feasibility of such an approach. Specifically, digital mediums are increasingly being used to deliver continuing professional development courses to lower-tier healthcare workers in Ghana, such as community nurses. Such approaches enable improved access to reliable training without relying on limited human resources. Beyond uptake, such approaches are both effective in increasing knowledge acquisition and acceptable to those receiving education, as evidenced by the successful remote training of formal providers to deliver a T2D self-management programme.^42^

Mobile phones have an important role in the delivery of remote training to community leaders. As established in the first study (Research stage 1 section), mobile phones are widely accessible resources with inherent use for information seeking in Ga Mashie. This suggests that positioning them as formal educational tools may be accessible, acceptable and feasible, as proposed in prior literature.^10,11,32–34^ Evidence in Ghana and other low–middle income contexts affirms that information provided through text or auditory messages and graphical illustrations is suitable for addressing informational gaps and increasing self-efficacy in managing T2D.^9,11^ Mobile devices have also been used to train community health workers on NCD management in low-resource settings using formats such as interactive modules with quizzes, video lectures, and downloadable resources.^40,41,43^ Similar devices and resources may also be feasible for upskilling community leaders.

#### Digital access to on-demand clinical guidance

The second level of support feasibly delivered to community leaders through mobile phones would be ongoing clinical guidance and supervision. Beyond initial training, community leaders would need on-demand access to reliable, case-specific advice to guide decisions on cases beyond the scope of their training.^45^ This guidance may relate to educational queries from the community or managing specific cases through either on-location basic treatment or referral to more specialised care.^40,42^ As mobile phones have already been used for clinical providers to provide community members^50–52^ and community health workers^46^ with advice with evidenced success, adapting such approaches to community leaders offers an easy route for establishing support pathways. Such an approach would present few community-based resource obstacles in their uptake, supporting their feasibility. However, they would require the effective integration of more experienced formal healthcare providers, which may present challenges in implementation if not effectively addressed.

NOP^46^ (see Networks of practice and other digitally enabled interventions in Ghana section) provides one opportunity. As NOP aims to scale-up services at the community level through formalising cross-tier collaboration networks,^49^ it emphasises the remote provision of information and support from experienced formal providers to healthcare workers in the community. By effectively integrating empowered community leaders in Ga Mashie into the NOP ecosystem, support and referral pathways between these providers and higher levels of care could be defined.

### Implications for future design

A particular observation highlighted in this research as well as prior literature is the need to ensure that digital health interventions are subordinate to the existing healthcare ecosystem. The preference of patients towards in-person, face-to-face interactions within healthcare due to their personal, trusted and secure nature^54^ is commonly observed but often overlooked. This is particularly relevant in collectivist cultures such as Ga Mashie, where human interaction is inherent within the social fabric of the community.^16^ Digital health interventions should aim to facilitate existing in-person services, rather than replace them, with such strategies having been observed as most likely to succeed.^12^ This research indicates that, rather than strategies that target individuals directly, digital health interventions may more effectively empower patients indirectly, by improving the capacity of the existing healthcare ecosystem to meet the needs of a population. Utilising existing and trusted leadership to collaboratively deliver digital health interventions with the community will build on relationships and leverage cultural and social norms. Success is likely to be mediated by alignment between community priorities, the priorities of the community leaders as well as those of the programme.^63^ Understanding alignment of these priorities in the cultural context of intervention implementation will be important when gauging the uptake and impact of any intervention.

As outlined in the background section, several previous digital interventions have been designed and deployed for use by individuals or representatives of the formal healthcare system. These have targeted either individual community members or recognised healthcare workers. A key insight from this study is that individuals are interconnected in various social structures, and that those social structures include trusted leaders who achieve that status through birthright (e.g. chief leaders), profession (e.g. pastors and teachers) or achieved status within a profession (e.g., ‘market queens’). These people have a potential role in promoting the wellbeing of their community, including educating on health conditions and facilitating access to formal healthcare. To realise this potential, digital tools can serve roles as described in the ‘A design sketch’ section.

While digital health presents exciting opportunities to innovate the healthcare context within resource-constrained settings, such innovation must be weighed against the user's comfort and preference for familiar and acceptable modes of care and risks such as data security and privacy. To achieve this, digital health designers should seek to drive digital innovation that mirrors interactions in the existing healthcare system rather than imposing potential solutions that radically challenge the healthcare status quo. This should ensure that digital innovations are acceptable and usable to target users, leading to greater acceptance and trust of digital innovation over time. It may then be possible to gradually push the boundaries of innovation, ensuring that the target users and their context are not isolated in the process.

### Limitations

There are inevitably limitations in a study that was as ambitious as this one, working with finite resources. The number of participants was determined by resource constraints (both time and person-power) while aiming to achieve representativeness across the variety of stakeholder groups (community members, healthcare workers, policy makers, etc.) involved in the study. Further, as the studies covered the rich aspects of people's lives and factors that shape their experience of and response to T2D, the use of digital technologies was not the primary focus of any of the sub-studies but featured in all the studies reported in this article.

In this mixed-methods study, the question of whether we achieved ‘data saturation’ is impossible to answer. Study 1 delivered a body of data from many participants (N = 98 + 39 + 30 = 167), all of whom addressed pertinent questions about roles of digital technologies in their lives or the healthcare system. We did not draw on a narrow body of data and interleave data gathering and analysis, through which saturation might have been achieved but at the cost of addressing a narrower research question. Rather, this study has sought to understand individual, community, health system and policy perspectives to identify barriers and facilitators for digital interventions addressing T2D in Ga Mashie. Our methods forced us to reflect on and challenge widespread assumptions about the roles of digital technologies in NCD management that a more narrowly focused study might have missed.

The studies reported here identified technical barriers and facilitators to deployment of a digital solution in this community. Because the workshop outcomes challenged our prior assumptions about the kind of digital intervention that might be appropriate, the refined ‘design sketch’ that involves empowering community leaders as first-line healthcare agents remains speculative and needs further research to both validate the proposal and flesh out the details.

### Future research

Since a digital approach was not the sole focus of the studies reported above, it would be valuable to engage the community further under an exclusively digital lens. It is crucial to evaluate the feasibility and acceptability of the proposed strategies amongst the community members and leaders for which these solutions would be designed. Further, research should identify specific information needs of community leaders to be able to effectively function as healthcare agents. Through such user-centred research, mobile interventions could be designed that empower community leaders to adopt roles as health agents in Ga Mashie.

While there are precedents for the proposed approach, in terms of related types of interventions, the specifics of this proposal are unique and raise questions that need to be addressed in future studies, including questions of ethics, sustainability, economics, data management and evaluation.

Ethical questions include whether empowering community leaders in the way proposed exacerbates existing power imbalances that could have negative consequences for community members. Counter to this is the fact that community leaders are generally highly trusted and respected. The community health worker literature suggests that trusted lay workers can provide a valuable bridge between community and the health system while acting as ‘brokers’ for both.^64,65^ Exploring the nature of this brokerage role and the autonomy of decision making within it may aid understanding of imbalances in power and potentially assist development of mitigation strategies. Recognition of the different forms of capital at play in the community and specifically how the symbolic capital and standing of leaders are leveraged for positive as well as potentially negative impact could also aid evaluation and understanding.^66,67^ Inevitably, trade-offs need to be made between power, accountability and responsibility, and these trade-offs need to be better understood. Conversely, acting as healthcare agents adds a burden of responsibility onto individuals in the community who may already have significant demands on their time and capacities.

Furthermore, a broader feasibility evaluation is required to ensure the sustainability of both digital interventions and the leaders’ integration as community health agents. Formal healthcare and policy stakeholders would need to be engaged in the design and implementation of mobile support tools to address potential barriers to implementation. This would include involving local providers in the generation of appropriate educational content and defining how the intervention can utilise existing infrastructure within the formal healthcare system. Furthermore, policy stakeholders should be consulted to ensure that community agents are effectively integrated into the health ecosystem by defining strategies for evaluation, reimbursement and ongoing support provision.

An essential facilitating factor would be economic viability of the proposed approach. While our studies to date have identified the economic burden to the individual,^68^ we have not conducted an economic evaluation of any proposed intervention. It will be important to conduct a cost-benefit analysis of a proposed digital solution once that solution has been validated and refined.

Further, such an intervention could only work if data is shared effectively between individuals and the various care providers they interact with. This naturally raises questions about what data is shared with whom and how, how data security is ensured and individuals’ privacy maintained. These questions are beyond the scope of the current study, but it would be essential to address them in a future study that follows this line of enquiry.

Every intervention design and deployment needs to be evaluated, and there are many important evaluation questions, ranging from safety and cost effectiveness through usability, feasibility and acceptability to clinical effectiveness. Different evaluation questions are important at different stages of design, development and deployment, and it is necessary to plan the relevant evaluation studies in advance of each stage.

Finally, while the proposed intervention focused on T2D in Ga Mashie, further research should explore the feasibility of adapting these digital strategies to other contexts. Specifically, if the proposed intervention can effectively empower Ga Mashie leaders as healthcare agents for T2D, it would be valuable to evaluate how to adapt this strategy to scale its impact. This includes exploring whether NCDs such as hypertension and obesity can similarly be addressed through the provision of basic education and testing services within the community. Furthermore, this includes evaluating the social structures in other communities in Ghana and SSA to explore the feasibility of delivering these services in other similar contexts. Through these explorations, these digital interventions may offer valuable strategies for addressing the growing burden of NCDs in SSA.

## Conclusion

This research demonstrates the need to explore beyond basic feasibility assessments when ideating digital innovations. As noted, literature exploring the challenges of digital health interventions has largely focused on individual-level barriers that influence the feasibility of implementation, such as an individual's access to digital resources and associated digital literacy. The users’ values that determine acceptance and uptake are positioned as secondary constraints or even ignored. This propagates the assumption that if these basic barriers are absent, one may effectively address healthcare challenges by designing digital tools for the individual. However, this research highlighted that despite a strong presence of individual-level facilitators such as access to mobile phones and their familiarity as information-seeking mediums, an individual-level education intervention is unlikely to be effective as it does not address the preferences and values of the target users.

Beyond feasibility, individual values crucially determine the acceptance of an intervention and its consequent effectiveness. Many digital interventions experience low utilisation despite their promise in terms of barriers and facilitators, suggesting that individual preferences are often overlooked in the design process. Effective digital health interventions are not only based in satisfying technical and clinical criteria but must also align with the health and interaction values of their intended users. It is crucial to actively engage the intended beneficiaries of an intervention to identify how a digital intervention may realise its impact. This seeks to re-ground the design process in the user, shifting away from the criteria of an intervention being technically feasible and rather ensuring that it is desired within the proposed context.

Based on our findings, we propose that digital tools could be used to empower community leaders to function as healthcare agents in their community. This positions community leaders as primary users who mediate the impact of the intervention on individuals in the broader community. Mobile phones, as ubiquitous information-seeking mediums, could be used to provide these leaders with the training and guidance required to deliver education and screening services at the community level. While an individual-level education intervention was identified as feasible, this system-level approach better supports the target community's needs and preferences towards in-person care and aligns with innovation trends within Ghana's healthcare ecosystem. Thus, this research challenges the preferential status assigned to individual-level digital health interventions, emphasising the importance of grounding design decisions in active engagement with the target beneficiaries and of supporting existing and familiar healthcare interactions. Through such approaches, one can ensure that digital interventions address core needs of the target community, resulting in such strategies moving beyond feasibility to effectiveness.

## Supplemental Material

sj-docx-1-dhj-10.1177_20552076251349705 - Supplemental material for Beyond individual barriers and facilitators: Digital interventions to address diabetes in urban Ghana Supplemental material, sj-docx-1-dhj-10.1177_20552076251349705 for Beyond individual barriers and facilitators: Digital interventions to address diabetes in urban Ghana by Ethan Gray, Ann Blandford, Samuel Amon, Publa Antwi, Vida Asah-Ayeh, Raphael Baffour Awuah, Leonard Baatiema, Sandra Boatemaa Kushitor, Hassan Haghparast-Bidgoli, Hannah Maria Jennings, Irene Akwo Kretchy, Daniel Strachan, Megan Vaughan and Edward Fottrell in DIGITAL HEALTH

sj-docx-2-dhj-10.1177_20552076251349705 - Supplemental material for Beyond individual barriers and facilitators: Digital interventions to address diabetes in urban Ghana Supplemental material, sj-docx-2-dhj-10.1177_20552076251349705 for Beyond individual barriers and facilitators: Digital interventions to address diabetes in urban Ghana by Ethan Gray, Ann Blandford, Samuel Amon, Publa Antwi, Vida Asah-Ayeh, Raphael Baffour Awuah, Leonard Baatiema, Sandra Boatemaa Kushitor, Hassan Haghparast-Bidgoli, Hannah Maria Jennings, Irene Akwo Kretchy, Daniel Strachan, Megan Vaughan and Edward Fottrell in DIGITAL HEALTH
