# Supplementary material for: Beyond individual barriers and facilitators: Digital interventions to address diabetes in urban Ghana
Source: Digit Health. 2025 Jul 28;11:20552076251349705. doi: 10.1177/20552076251349705 (PMC12304655; doi:10.1177/20552076251349705)
Supplement: sj-docx-1-dhj-10.1177_20552076251349705 - Supplemental material for Beyond individual barriers and facilitators: Digital interventions to address diabetes in urban Ghana [file sj-docx-1-dhj-10.1177_20552076251349705.docx]

# Supplementary material: focus group and interview guides

## CARE diabetes Ghana - Community members focus group discussions

Three versions of this guide were developed for males, females and youth. We include just the youth guide as an illustration. Those for males and females adapted the examples to be relevant to the sex of participants.

**CARE Diabetes Ghana - Community members focus group discussion (youth group 18–24-year-olds)**

**++++++++++++++++++++++++++++++++++++++++++++++++++++++++++++++++++++++++++++++++++++++++++**

**For researchers, you will need:**

- A quiet space to meet to discuss the topics with 6-8 respondents
- To follow the process for attaining all respondents informed, written consent to participate (including to record the discussion) before commencing
- Have dispersed all refreshments or indicated when they will be dispersed prior to commencing
- All participants must wear their disposal mask and sanitize their hands prior to the start of the FGD
- Observe social/physical distance in the sitting arrangements and interaction during the focus group discussion

**++++++++++++++++++++++++++++++++++++++++++++++++++++++++++++++++++++++++++++++++++++++++++**

**At the beginning of the discussion the researcher should:**

- Introduce themselves and ask participants to do so too
- Thank everyone for attending and for agreeing to participate in the discussion
- Confirm informed consent and recording of the session
- Explain the purpose of the discussion (objectives/introduction)
- Explain that people are free to contribute as they feel comfortable and that there are no wrong or right answers – we are interested in your own opinions and experiences based on those opinions
- Collect biodata of all participants

**++++++++++++++++++++++++++++++++++++++++++++++++++++++++++++++++++++++++++++++++++++++++++**

**Introduce**: The aim of the group discussion, the importance of confidentiality and the task itself

| **Say:** | The aim of our meeting today is to build a story of two friends in your community, a young man and woman. They are not real but we want to imagine that their experience as young adults in Ga-Mashie is real and could happen in your community. It is important that we keep the names of real people and stories private in this discussion. While we can draw on these experiences in our discussion, we wish to respect all of your privacy and that of other members of the community. |
| --- | --- |
| **Discuss:** | The task and any questions |
| **Say:** | Today I want to introduce you to **Nii** and **Naa.** We can give them different names if you would prefer (*ask and agree another name*). Nii is 22 and Naa is 20 years old. They have both lived in Ga-Mashie their whole lives and are childhood friends. They are [occupation/studying]. Nii has recently been diagnosed with type 2 diabetes. |

**++++++++++++++++++++++++++++++++++++++++++++++++++++++++++++++++++++++++++++++++++++++++++**

1. **Ask**: Tell us about what an average day looks like for Nii and Naa? What do they do? Where do they go?

| **Discuss:** | We want to understand their lifestyle including what and how they eat (types of meals – bought or not), when they wake up, their routine, what kind of work (if relevant), are they are active/not active, how/when they move around. How much time do they spend using their phones, and what for? Is this different for men and women? |
| --- | --- |
| **Probe:** | Explore everyday habits of young people in Ga-Mashie. What are their daily habits? - are any of these risk factors for diabetes? This can include physical inactivity, ‘unhealthy’ diet (i.e. fried foods etc), smoking, drinking etc |
| **Please explain:** | Nii and Naa’s life and the way they live in Ga Mashie. |

**++++++++++++++++++++++++++++++++++++++++++++++++++++++++++++++++++++++++++++++++++++++++++**

1. **Say/Ask**: What things do Naa and Nii do to socialise with their friends? Are there community activities that they are able to get involved in? What sort of activities are these? What activities do you think they would like to get involved in? Is there anything that could prevent them i.e. time, popularity, whether it is seen as important, from getting involved? How could digital communication help encourage them to engage?

| **Discuss:** | Day activities and night activities. Lifestyle - The role of group activity/ participation for young people. Is it important? What things are done when socialising – drinking, eating etc. Are there existing activities that young people engage in? Is any of this linked to health improvement? Is there a role for digital technology? |
| --- | --- |
| **Probe:** | What encourages young adults’ participation in activity in the community? What helps and hinders their involvement? Would reaching and encouraging them through their phone or other new technologies might help (and if so, what sort of content would be ideal – e.g. information, social media, stories, others?) |
| **Please explain:** | What would encourage young adults to live healthier lifestyles and participate in group activities |

**++++++++++++++++++++++++++++++++++++++++++++++++++++++++++++++++++++++++++++++++++++++++++**

1. **Say/Ask**: What things do you think Naa and Nii like to eat? Where do they get food from?

| **Discuss:** | Diet – what kind of foods are typically eaten? What do they think of the food? How often do they eat the meals they eat? What are their views around food and diet? Is it important or not? Similarities and differences to parents and family? |
| --- | --- |
| **Probe:** | What are the typical foods they might like to eat? What do they like about them? What is important when it comes to the meals they eat – taste, nutritional value etc? What is important when it comes to food – convenience, price, availability etc? |
| **Please explain:** | What food do young people like to eat and what do they think about the food available in Ga-Mashie? |

**++++++++++++++++++++++++++++++++++++++++++++++++++++++++++++++++++++++++++++++++++++++++++**

1. **Say/Ask**: Now we have talked a little about what Naa and Nii typically do, do you think they would consider their lifestyle to be healthy? Why/why not? Thinking about how Naa and Nii lead their lives, do you think their lifestyle is healthy? Why/why not?

| **Discuss:** | Risk factors of acquiring diabetes |
| --- | --- |
| **Probe:** | Key to probe differences between Nii and Naa remembering that the participants are building a profile of these fictional characters. Thereafter, understanding of risks of diabetes, if raised by respondents, probe areas around diet (especially sugar, salt, and oil consumption), exercise vs sedentary lifestyles, body size (i.e. being overweight), stress, tobacco and alcohol consumption and environmental influences. How living in Ga-Mashie itself influences the risk of diabetes. Day time built environmental influences and night-time environmental influences. Food, alcohol, physical activity spaces, community layout and how it enables or inhibits health promoting behaviours |
| **Please explain:** | Opinions on the risks of acquiring diabetes – what may contribute to having it? |

**++++++++++++++++++++++++++++++++++++++++++++++++++++++++++++++++++++++++++++++++++++++++++**

1. **Say/Ask**: Nii was recently diagnosed with diabetes. What do you know about diabetes and how do you think it affects his life? What do you think the causes of diabetes are? Why do you think he has diabetes? What makes people at risk of developing diabetes? Do you think this affects his family? If so, how?

| **Discuss:** | The impact of having diabetes on Nii’s life and on the family |
| --- | --- |
| **Probe:** | Awareness of diabetes among the group, what living with diabetes means, attitudes towards people living with diabetes and any stigma there may be about it. Challenges that people living with diabetes might face in Ga Mashie (probing for financial, geographic, and social access.) Have there been changes to the way the family lives due to his diagnosis? What has the impact been like – negative/positive? |
| **Please explain:**  **Please note:** | Awareness of diabetes and what living with diabetes would be like for someone like Nii’s in Ga Mashie.  Some FGD participants may know people who live with diabetes/be diagnosed themselves but may not wish to disclose this. Please do not directly ask about diabetes status/family members diagnosis but do explore insights of people that are happy to discuss it. |

**++++++++++++++++++++++++++++++++++++++++++++++++++++++++++++++++++++++++++++++++++++++++++**

1. **Say/Ask**: What services and supports does he need? How available and accessible is this support and services to Nii and are they good? What makes these services good or not? What makes it easy and challenging for Nii to access these services?

| **Discuss:** | The supports and services, including medications, Nii’s needs |
| --- | --- |
| **Probe:** | Awareness of supports and services available and needed for diabetes care and what might enable and constrain access to them, probe formal and informal health providers [ADD APPROPRIATE NAMES], community or social groups at the community, and what determines quality |
| **Please explain:** | The range of available supports and services – both formal and informal - in Ga Mashie for someone like Nii who lives with diabetes |

**++++++++++++++++++++++++++++++++++++++++++++++++++++++++++++++++++++++++++++++++++++++++++**

1. **Say/Ask**: Naa did not know that much about diabetes before she found out Nii was diagnosed. Do other young people in Ga Mashie know about diabetes? Where in Ga Mashie would she be able to get more information about diabetes? Where do other people in the community get information about diabetes and other non-communicable diseases from?

| **Discuss:** | Sources of health information in Ga Mashie, how accessible it is and what makes people trust it, the role of digital sources of information now and with potential for positive impact |
| --- | --- |
| **Probe:** | Phone use for information that is trusted, digital content of different types, healthcare professionals (within government health system and also informal providers), family, friends and what enables easy access and what determines quality and reliability. |
| **Please explain:** | Information sources (big focus on availability and trust of digital sources of information) |

**++++++++++++++++++++++++++++++++++++++++++++++++++++++++++++++++++++++++++++++++++++++++++**

1. **Say**: What else is important for us to know about living with diabetes in Ga Mashie?

| **Discuss:** | What respondents think is important and we may have missed in our discussions so far |
| --- | --- |
| **Probe:** | Beliefs around how things do happen and how things should happen |
| **Please explain:** | You are the experts in what happens in your community, please do help us understand |
|  |  |

**++++++++++++++++++++++++++++++++++++++++++++++++++++++++++++++++++++++++++++++++++++++++++**

**Thank respondents for their time and stop recording**

**++++++++++++++++++++++++++++++++++++++++++++++++++++++++++++++++++++++++++++++++++++++++++**

**Additional points for researchers:**

- Often participants will want to talk to you about the research topic after the group discussion has officially ended (and the recording has stopped). Please do try and answer their questions as best you can and also take notes of any additional points made as well as observations about the group, their dynamics and what may have influenced their perspectives in your view. You can add these notes to the transcriptions of the recording – they will be really valuable for the analysis!

**++++++++++++++++++++++++++++++++++++++++++++++++++++++++++++++++++++++++++++++++++++++++++**

STUDY 2- **Interview guide for healthcare providers**

**For researchers**

At the beginning of the discussion the researcher should:

- Introduce themselves and ask participants to do so too
- Thank respondent for attending
- Explain the purpose of the discussion (objectives/introduction) and what the respondent’s participation will involve
- Explain that people are free to contribute as they feel comfortable and that there are no wrong or right answers, and that we are interested in your own opinions and experiences based on those opinions
- Let them know the average time the interview is likely to span
- Attain the informed consent of the respondent to participate and have information recorded and have the discussion recorded

**+++++++++++++++++++++++++++++++++++++++++++++++++++++++++++++++++++++++++++++++**

1. **Type 2 diabetes knowledge and perceived risks**

**Scenario for discussion:** Suspected cases of diabetes (hyperglycaemia)

**Say:** A community member (Mr. Nortey, a fisherman aged 57) presents to you in your facility or care point feeling unwell and says he does not have much energy. Based on your preliminary examinations, you suspect the person is having hyperglycaemia.

**Ask (all):**

- What is your understanding of hyperglycaemia? If respondent is unsure explain that Mr Nortey has low energy and is sick (e.g. potentially if respondent is a herbalists, traditional medical practitioner, faith healers or itinerant healthcare provider)

**For herbalists, traditional medical practitioner, faith healers or itinerant healthcare provider respondents only, ask:**

- What is diabetes? What impact does diabetes have in the community?

**Probe:** FWs to encourage explanation and examples

**Ask (all):**

- Is Mr Nortey a typical patient who would present to you/your facility with these symptoms? Who else is likely to report with diabetes/hyperglycaemia, other health conditions, to you/ your facility?

**Probe:** gender, age, social class, education level, local residents or commuters/ non-residents

- What do you think are the key risks for developing diabetes (i.e. type 2 diabetes)?

**Probe**: Ask for elaboration, examples/stories and additional risks (i.e. respondents should be invited to come up with multiple risks)

- Could you please describe how you provide diabetes care services to someone such as Mr Nortey? Ask whether it changes depending on whether Mr. Nortey knows he has diabetes?

**Probe**:

- Diagnosis – What kinds of services do you/ facility provide to help with initiating or diagnosing diabetes? How do you go about helping such a person? What if someone has not been diagnosed but appears to have diabetes – what do you do? Please elaborate
- Management - the processes that are in place to care for patients who are diabetic or at risk of diabetes? (i.e. are including whether; referrals are made? Is and if so, to whom and with what follow up/tracking?
- Prevention – what is done to encourage behaviours that reduce likelihood of diabetes onset or reduce risks associated with having diabetes
- Whether medication is offered? Are alternatives to medicine offered? Are other therapies/treatments are provided and other information regarding the type of care is received by the patient?
- What care is provided when a patient has multimorbidity (i.e. more than one illness or condition)?
- How do you manage emergencies and complications related to diabetes? Can you please explain while providing examples (*NB: CAUTION FOR FWs: without revealing the identity of a patient*)?

1. **The nature of care provided: performance priorities, community trust and challenges and opportunities**

**Say:** Treatment of chronic conditions such as diabetes is complex and different factors influence the nature of care provided. Different care providers provide different diabetes care and care varies. We are interested in what you do and think is important. There are no correct answers – we just want to know what you think.

**Ask:**

- How do you judge the quality of the care you provide? What do you base this judgement on? Why? Please tell me more.

**Probe**: FWs to encourage explanation and examples

- What impact does your approach to diabetes care and management have in the community?

**Probe**: FWs to encourage explanation and examples

- What do you start with or prioritise when it comes to diabetes care and prevention? What is the most important thing to say and do with a patient that has diabetes or you suspect has diabetes? What do you base this on?

**Probe**: FWs to encourage explanation and examples

- What helps you provide quality diabetes care and prevention services? What are the main opportunities for providing useful and needed care and prevention services?

**Probe**: FWs to encourage explanation and examples

- What can prevent you from providing quality diabetes care and prevention services? What are your biggest challenges?

**Probe**: FWs to encourage explanation and examples and encourage respondents to link the challenges they name to diabetes care and prevention

- What training would help you with the quality of diabetes care and prevention services you provide? Would you like this training? What training would help other service providers?

**Probe**: FWs to encourage explanation and examples

- What other supports, beyond training would help your provide quality diabetes diagnosis, care and prevention services?

**Probe:** supervision, supplies and commodities, workload, other, with explanation and examples

- How do others judge the quality of the diabetes care and prevention services you provide? How is your performance judged/audited?  How are the diabetes services of other providers judged/audited?

**Probe:** by your employer, by the community, by people seeking your services, by colleagues, with explanation and examples.

- Do people that seek your services trust you? Do people in the community trust your advice when it comes to diabetes and other health challenges? Please explain why/why not? What does trust depend on? Is it anything to do with diabetes specifically? How is trust earned? Do other providers have the trust of the community? Which ones and why?

**Probe:** FWs to encourage explanation and examples

1. **Perceptions of the impact of living with diabetes (known and unknown)**

**Say:** We would now like to talk to you about what you think the impact of having diabetes has on people in this community. There are no correct answers, we are interested in your opinion.

***Instructions for FWs:*** *remember to draw out the respondents by asking them to tell you more and inviting them to give examples and tell stories.*

**Ask:**

- What do you think is the main impact living with diabetes has on people in the community? How does it vary and what does this variation depend on (*e.g. probe for variation by gender, age, lifestyle, profession, other*)?
- Does the impact of living with diabetes depend on whether it is diagnosed? Why/why not? Please explain
- For people in this community, please tell us about:
- The Economic burden of living with diabetes (i.e., issues relating to finances)

**Probe:** FWs to encourage explanation and examples

- Psychological burden of living with diabetes (i.e., issues relating to how people think and feel and how this influences how they act)

**Probe:** FWs to encourage explanation and examples

- Social burden of living with diabetes (i.e., issues relating to how people interact with family, friends and community members)

**Probe:** FWs to encourage explanation and examples

- Physical Burden (health) i.e., condition-related burden of living with diabetes (i.e., issues relating to modified or changed physical abilities)

**Probe:** FWs to encourage explanation and examples

- Therapy-related burden of living with diabetes (i.e., issues relating to seeking and accessing appropriate and valued care)

**Probe:** FWs to encourage explanation and examples

1. **The role of digital and community-based approaches to diabetes care and prevention and collaboration with other disciplines and providers**

**Say:** We are interested in understanding how the use of digital tools and communication (e.g. smartphones) as well as community activities may help with diabetes care and prevention. We are also interested in how different health providers work together and also work with other groups in diabetes prevention and care

**Ask:**

- How is information gathered on people living with diabetes?  What types of patient registries are used? Which digital tools are used for capturing data?

**Probe**: FWs to encourage explanation and examples

- How are digital tools used in your work? Probe: smartphones, tablets, other.

**Probe**: FWs to encourage explanation and examples

- What community based approaches exist to prevent and care for those living with diabetes? Are there support systems in the community for diabetes control and management? If yes, what are they? How can patients access these services? If no, would this be beneficial?

**Probe**: FWs to encourage explanation and examples

- What tools and support systems are available to promote community-led response for diabetes care such as regular blood glucose tests and guidance on medicine use, dietary control, and physical exercise provided by primary health institutions (PHIs)? Are they effective? Why or why not? How could these tools be better used for optimal diabetes care?

**Probe**: FWs to encourage explanation and examples

- Do you work with other health workers in the prevention and management of diabetes? Please say who (in terms of roles, not personal names)

**Probe**: FWs to encourage explanation and examples around what is happening currently, how they envisage working with other members of the healthcare team, integration of traditional and herbal medical practice, who other stakeholders are and their roles

1. **Community Health Care Workers**

**Say**: We are interested in understanding what you know about and your opinion of community health workers in your community.

**Ask**:

- What are/is are the roles and expectations of CHWs in diabetes care provision from the health facility/managers, community/service users, and CHW perspectives?
- What do CHWs think about the healthcare delivery expectations of them with regard to whether they:
- Play important roles in health service delivery
- have the requisite capacity to deliver care
- Acceptable by their community
- What do CHWs think about the community expectations of them with regard to whether they are:
- Play an important role
- Feasible to accommodate
- Acceptability to them
- What are the capacity needs of CHW in diabetes care? Ask when they last received any training for diabetes care
- What do CHWs feel are their most important role performance priorities? To what degree do these differ from programmatic priorities?
- Do you use any digital tools to support your work? Do you recommend any to your patients? Are there any digital tools that would help you in your work?
- Explore CHWs role in the home-based care and support for people diagnosed with diabetes i.e. monitoring, optimal medicines utilization, reminders for hospital appointments etc

**+++++++++++++++++++++++++++++++++++++++++++++++++++++++++++++++++**

**Thank respondents for their time and stop recording**

**+++++++++++++++++++++++++++++++++++++++++++++++++++++++++++++++++++++++++++++++**

**Additional points for researchers:**

- Often participants will want to talk to you about the research topic after the group discussion has officially ended (and the recording has stopped). Please do try and answer their questions as best you can and also take notes of any additional points made as well as observations about the group, their dynamics and what may have influenced their perspectives in your view. You can add these notes to the transcriptions of the recording – they will be really valuable for the analysis!

**+++++++++++++++++++++++++++++++++++++++++++++++++++++++++++++++++++++++++++++++**

**Interview guide for industry, policy makers, CSOs and government**

**For researchers**

At the beginning of the discussion the researcher should:

- Introduce themselves and ask participants to do so too
- Thank everyone for attending and for agreeing to participate in the discussion
- Confirm informed consent and recording of the session
- Explain the purpose of the discussion (objectives/introduction)
- Explain that people are free to contribute as they feel comfortable and that there are no wrong or right answers – we are interested in your own opinions and experiences based on those opinions

**Questions**

1. What specific policies, programs and initiatives are in place to prevent and control diabetes?  Probe for specific programs and policies on:

- Reduce tobacco use
- Reduce harmful use of alcohol.
- Promoting physical activity.
- Promoting healthy diet.

1. Are there any contextual facilitators influencing the development and implementation of interventions or policies to prevent and control diabetes in line with the WHO Best Buys.?

Probes:?

1. Are there any contextual barriers influencing the development and implementation of interventions or policies to prevent and control diabetes in line with the WHO Best Buys.?
2. Describe the governance structure supporting national T2D response.

Probes: ?

1. What programs and policies exist at the national or local levels to increase awareness and strengthen the knowledge base on the magnitude and nature of NCDs/diabetes to be specific, risk factors as a public health concern- awareness programmes, improved monitoring, and surveillance systems.
2. Are existing policies and interventions doing enough to provide consumers with accurate information on food and beverage products as part of measures to reduce their NCDs risk especially overweight, obesity, and diet-related NCDs? Probe to unearth implementation challenges associated with such policies and measures among regulatory bodies.
3. What health systems measures are in place to prevent and control diabetes at the basic primary health care package with referral systems to all levels of care to advance the universal health coverage agenda?
4. Are there any health financing mechanisms to support diabetes prevention and control?
5. Are there some systems or measures in place at the various levels of care to

- support early detection, screening, treatment and management of diabetes?
- train the health workforce and strengthen the capacity of health systems, particularly at the primary care level, to address the prevention and control of diabetes?
- improve the availability of the affordable basic technologies and essential medicines, including generics, required to treat major diabetes, in both public and private facilities?
- develop and implement a national multisectoral policy and plan for the prevention and control of diabetes
- prevent foot care for people with diabetes (including educational programmes, access to appropriate footwear, multidisciplinary clinics),
- prevent diabetic retinopathy
- support people in self-management using tools such as blood glucose monitors

**Probe to find out whether each of the above systems or measures work or are well implemented.**

1. Does the current NCD policy make sufficient provision for the prevention and control of diabetes?
   - 1. What is the place of diabetes prevention, screening and treatment services in the current NCD policy?
     2. What specific activities and interventions are targeted by the policy for diabetes prevention and control?
     3. Which stakeholders are involved and what will be their roles?
2. Can you share with us information about policies and programs to

- Raise public and political awareness, understanding and practice about prevention and control of diabetes
- Integrate diabetes into the social and development agenda and poverty alleviation strategies
- Adoption of front-of-package warning labelling to tackle NCDs in Ghana
- Implementation of nutrition education strategy (food-based dietary guidelines and nutrition information table on the back of food packaging) for preventing NCDs.
- Strengthen international cooperation for resource mobilization, capacity-building, health workforce training and exchange of information on lessons learned and best practices
- Engage and mobilize civil society and the private sector as appropriate and strengthen international cooperation to support implementation of the action plan at global, regional and national levels
- Prioritize and increase, as needed, budgetary allocations for prevention and control of diabetes
- Develop and implement a prioritized national research agenda for diabetes
- Prioritize budgetary allocation for research on diabetes prevention and control
- Make more (or less) use of healthcare data and analytics in care management
